# Copper selective 8-aminoquinoline based tetradentate chelators as anticancer agents

**DOI:** 10.1039/d4md00171k

**Published:** 2024-06-04

**Authors:** Yingzhen Guan, Michel Nguyen, Anne Robert, Yan Liu, Bernard Meunier

**Affiliations:** a School of Chemical Engineering and Light Industry, Higher Education Mega Center, Guangdong University of Technology (GDUT) Guangzhou 510006 P. R. China yanliu@gdut.edu.cn; b Laboratoire de Chimie de Coordination du CNRS 205 Route de Narbonne 31077 Toulouse Cedex 4 France bernard.meunier@lcc-toulouse.fr

## Abstract

Cancer cell proliferation and metastasis are known to be dependent on angiogenesis which is regulated by several parameters including copper availability. Tetradentate monoquinoline (TDMQ) ligands constitute a series of chelators tailored to regulate copper homeostasis due to their specificity for copper(ii) with respect to Cu(i) or other biometals like iron or zinc. One of these chelators, TDMQ20 efficiently inhibits both proliferation and migration of several human cancer cell lines, better than the reference drug 5-fluorouracil, and with higher selectivity indexes with respect to non-cancer human cells. The biological activity of TDMQ20 may be driven by the coordination chemistry of copper, and the ability of this chelator to restore copper homeostasis and its subsequent redox properties. The anticancer mechanism of action of TDMQ20 involves intracellular production of reactive oxygen species, drastic mitochondrial damages and induction of tumor cell apoptosis. These data support the selection of TDMQ20 as drug-candidate against several human cancers.

## Abstract 2

The copper chelator TDMQ20 inhibits proliferation and migration of cancer cells. Its mechanism, maybe driven by the coordination chemistry of copper, involves intracellular production of reactive oxygen species and induction of tumor cell apoptosis.

## Graphical abstract

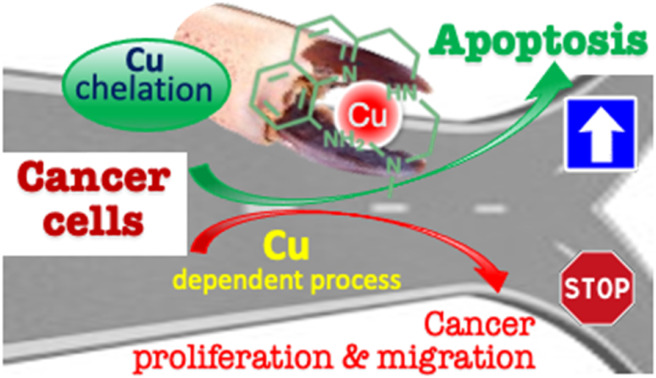

## Introduction

Cancer is the second leading cause of death after cardiovascular diseases worldwide. Many different methods of cancer treatments have been developed, including surgery, radiation therapy, chemotherapy, gene therapy and immunotherapy. Because of the complexity of cancer and the capacity of cancer cells to create resistant strains, there is a recurrent necessity to create new tools to fight resistance, including new chemical agents.^1–3^ Among all different approaches that have been developed in chemotherapy, metal ligands have not been extensively explored, despite the fact that it has been known for a long time that the content of metal ions can be different in cancer cells compared to normal ones. For example, the concentration of copper ions in metastatic carcinoma and in malignant glioma is 1.5 and 1.3 times higher than that of controls, respectively.^4^ Copper ions are essential for metastasis proliferation.^5^ It should be noted that the majority of deaths (at least 2/3) due to solid cancer tumors are in fact caused by metastases. In this context, the development of new drugs able to control metastatic processes is an essential strategy to reduce cancer mortality.^6^

**Fig. 1:**
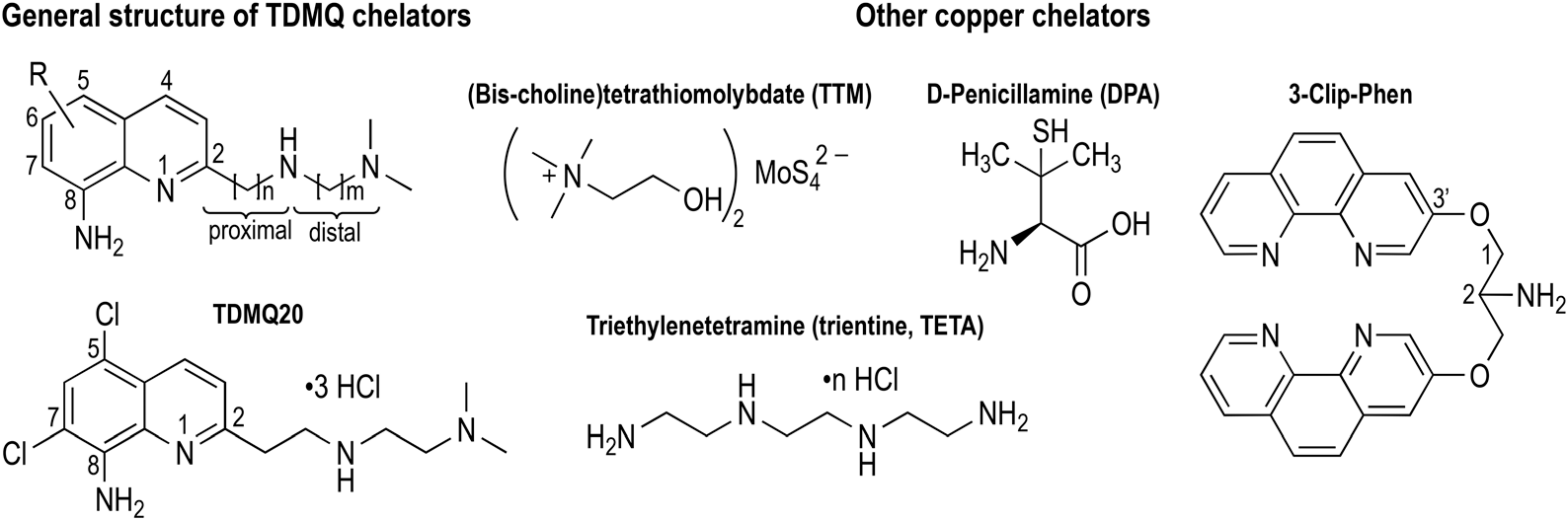
Structures of TDMQ specific Cu(ii) chelators, and of some other Cu chelators considered for antiangiogenic properties.

Copper is involved in angiogenesis^7,8^ and, consequently, in tumour growth and dissemination (metastasis) of a large variety of cancer cells,^5,9,10^ conversely, depletion of copper has been shown to inhibit angiogenesis in a wide variety of cancer cell and xenograft systems.^11^ More generally, cellular uptake of copper has recently been shown to play a central role in inflammation and cancer progression through a general mechanism involving regulation of cell plasticity.^12^ Consistently, assays in xenograft mice,^13^ and some anticancer clinical trials have been performed with copper chelators which are currently used for the treatment of Wilson's disease, such as d-penicillamine (DPA) or trientine (TETA), or which have been studied for this application, such as (bis-choline)tetrathiomolybdate (TTM) (Fig. 1). However, these attempts have not been very successful due to the lack of metal specificity of these ligands and/or to their potential toxicity.^11,14–17^ We decided to re-investigate the activity of copper ligands, nearly 20 years after one of us reported the antiproliferative activity of copper chelators based on a clip-bis-phenanthroline motif, against L1210 cells.^18^ As a matter of fact, the actual challenge is to develop efficient and highly selective copper chelators as anticancer agents that will be able to inhibit not only cell proliferation but also angiogenesis, without significant accumulation and toxicity against normal human cells.

We initially developed a series of specific copper(ii) tetradentate ligands named TDMQ (Fig. 1), as potential agents for the treatment of Alzheimer's disease (AD), since it has been evidenced that the homeostasis of copper is disturbed in the brain of patients with AD.^19–21^ TDMQ ligands were designed to provide chelation of Cu(ii) using the two aromatic nitrogen atoms of a 8-aminoquinoline nucleus, and two nitrogen atoms of the modular side chain attached at C2 of the quinoline moiety. By tuning the side chain length (*n* = *m* = 2, Fig. 1), TDMQ offered a tetradentate nearly perfect square-planar coordination sphere around the copper(ii) ion, generating N4 complexes with a 1/1 metal/ligand stoichiometry. For this reason, several TDMQ chelators such as TDMQ20 (Fig. 1) are highly selective for Cu(ii) with respect to Cu(i), and to other biometals such as Fe^III^ or Zn^II^, or biologically important divalent cations (such as Ca^2+^ or Mg^2+^).^21,22^ As suggested by one referee, we investigated the chelation of Fe^II^ by TDMQ20, and we found that the affinity of this chelator for Fe(ii) was very low, in fact below values measurable under standard UV-visible conditions (see below). Moreover, these chelators do not disturb the biological activity of essential and ubiquitous copper enzymes such as Cu,Zn-superoxide dismutase.^22^ This specificity and the resulting lack of redox activity of the Cu(ii)TDMQ20 complex is responsible for its ability to reverse the Cu-induced pathology in the brain of mice with Alzheimer's disease, without any acute or long-term toxicity.^23^

Because of the suitable pharmacological profile,^24^ we decided to explore the possibility of using TDMQ20 to induce selective cancer cytotoxicity by reducing copper supply of tumors. We therefore evaluated the cytotoxic and antiproliferative activities of TDMQ20 against several cancer cell lines, using 5-fluorouracil (5-FU) as comparator. TDMQ20 was found highly cytotoxic against non-small cell lung carcinoma (A549), cervix cancer HeLa and hepatocarcinoma HepG2 cell, exhibiting IC_50_ values ranging from 14 to 16 μM *in vitro*, at lower doses than those observed for the reference drug 5-fluorouracil, and also with higher selectivity indexes with respect to non-cancer human cells. TDMQ20 also exhibited a significant antiproliferative activity on HeLa cells *in vitro*. The mechanism of its cytotoxicity and antiproliferative activity involve intracellular production of reactive oxygen species, drastic mitochondrial damages and induction of apoptosis.

## Results and discussion

### Titration of TDMQ20 by Fe^II^(SO_4_)_2_(NH_4_)_2_·6H_2_O

The titration of TDMQ20 (20 μM) was achieved in Hepes buffer, pH 7.4, and monitored by UV-vis. Spectrometry, as reported in ref. 21, except that TDMQ20 and Fe(ii) salt solutions were carefully degassed and kept under argon. After addition of 20 or 30 molar equivalents of Fe(ii) with respect to TDMQ20 (corresponding to 400 or 600 μM of Fe in the cuvette, respectively), the absorbances of TDMQ20 were unchanged, at *λ*_max_ = 258 and 345 nm (Fig. 3a and b, respectively). Moreover, the spectrum of the mixture of TDMQ20 + 20 or 30 mol. equiv. of Fe(ii) (Fig. 3, blue traces) was superimposable to the spectrum of TDMQ20 (red traces) + the spectra of Fe(ii) 400 μM (Fig. 3a) or 600 μM (Fig. 3b) (green traces). This result attests the lack of significant Fe(ii) coordination by TDMQ20 in these conditions. From these data, the log *K*_app_ [Fe(ii)-TDMQ20] can be evaluated as below 3.^21^

### Pharmacological activity of TDMQ ligands on different cancer cell lines

#### Cytotoxicity to cancer cells, selectivity with respect to non-cancer cells

The viability of several cancer cell lines was evaluated *in vitro* using the MTT assay, assessing cell metabolism through the activity of NAD(P)H-dependent cellular oxidoreductases. This test is based on the reduction of a colourless tetrazolium salt by mitochondrial succinate dehydrogenase to produce the violet formazan derivative that is quantified by UV-visible spectrometry (*λ*_max_ = 570 nm), thus reflecting the number of viable cells present.

**Fig. 2:**
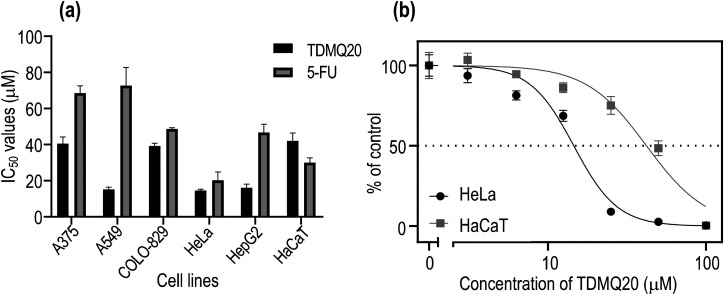
(a) IC_50_ values of TDMQ20 and 5-FU on several cancer cell lines, and on HaCaT cells. (b) Proportion of viable cancer HeLa and non-cancer HaCaT cells with respect to concentration of TDMQ20 (log scale). The bars represent SD values of at least three independent experiments.

The cytotoxicity of TDMQ20 was evaluated up to 100 μM after a 48 h incubation period, on the human cancer cell lines, metastatic melanoma A375, non-small cell lung adenocarcinoma A549, melanoma COLO-829, cervical cancer HeLa, and hepatocellular carcinoma HepG2. The human immortalized keratinocyte cell line HaCaT was used as non-cancer reference cell line, and the clinically used anticancer drug 5-fluorouracil (5-FU) was used as a comparator. Results are reported in Fig. 2 and Table 1.

**Table 1:** Viability determined by the MTT assay, of cancer cell lines (metastatic melanoma A375, non-small cell lung adenocarcinoma A549, melanoma COLO-829, cervical cancer HeLa, hepatocellular carcinoma HepG2) compared to the human immortalized keratinocyte cell line HaCaT, treated with TDMQ20 or 5-FU for 48 h. IC_50_ are mean values ± SD (μM) of at least three independent experiments. Selectivity index SI stands for IC_50_ (HaCaT)/IC_50_ (cancer cell)

Drugs/cell lines	IC_50_ values (μM) ± SD at 48 h					
	Metastatic melanoma A375	Non-small cell lung ADC A549	Melanoma COLO-829	Cervical cancer HeLa	Hepatocell. carcinoma HepG2	Human immortalized keratinocyte HaCaT
TDMQ20	38.1 ± 7.0	**16.2** ± 2.9 SI = 2.3	40.2 ± 4.9	**14.5** ± 0.8 SI = 2.8	**16.6** ± 4.7 SI = 2.3	41.1 ± 4.8
5-FU	68.5 ± 4.0	72.6 ± 10.0 SI < 1	48.7 ± 0.7	20.3 ± 4.6 SI = 1.5	46.7 ± 4.7 SI < 1	30.0 ± 2.7

**Fig. 3:**
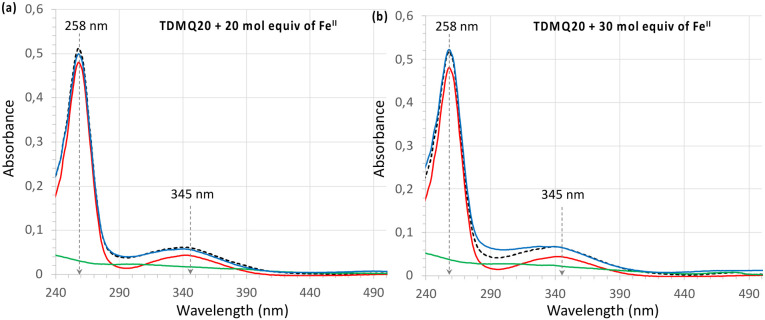
UV-visible spectra of TDMQ20 20 μM alone (red trace) or after addition of (a) 20 mol equiv. or (b) 30 mol. equiv. of Fe(SO_4_)_2_(NH_4_)_2_·6H_2_O, corresponding to 400 or 600 μM, respectively, (blue trace). The dashed black traces stand for the arithmetic addition of the spectrum of TDMQ20 + spectra of Fe(SO_4_)_2_(NH_4_)_2_·6H_2_O, (a) 400 or (b) 600 μM alone. The spectra of Fe(SO_4_)_2_(NH_4_)_2_·6H_2_O alone (a) 400 or (b) 600 μM (green traces), are given for comparison.

In all cases, cell viability was dependent on the concentration of TDMQ20, as depicted in Fig. 2b for HeLa cells. The anticancer activity of TDMQ20 was quantified as the concentrations of drug inhibiting by 50% the growth of each cell line (IC_50_, Fig. 2a, Table 1).

TDMQ20 exhibited higher cytotoxic activities (lower IC_50_ values) than 5-FU against all the tested cancer cells. Its cytotoxic activity is especially high against the non-small cell lung carcinoma A549, the cervix cancer HeLa cells and the hepatocarcinoma HepG2, with IC_50_ values in the range 14–16 μM. The cytotoxicity of TDMQ20 on the non-cancer cell line HaCaT (IC_50_ = 41 μM) was lower than that of the reference drug 5-FU (IC_50_ = 30 μM). The selectivity [SI = IC_50_ (HaCaT)/IC_50_ (cancer cell)] of TDMQ20 for A549 cells, HeLa cells and HepG2 cell lines with respect to non-cancer cells HaCaT was ranging from 2.5 to 2.8. This is depicted in Fig. 2b for HeLa cells. Such safety window is better than that of 5-FU.

#### Antiproliferative activity of TDMQ20 against HeLa cells. Colony-forming assay

**Fig. 4:**
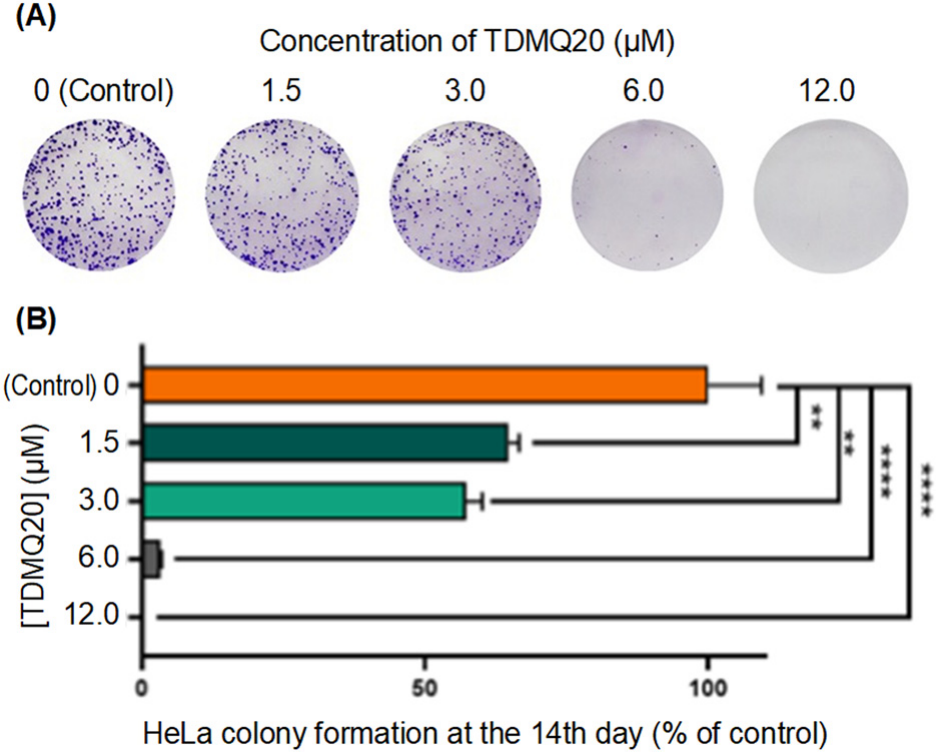
(A) Images of HeLa cells treated for 14 days with TDMQ20 at concentrations 0 (control), 1.5, 3.0, 6.0 or 12.0 μM, after staining by crystal violet. Images of a representative experiment. (B) Histograms of the cell counts of HeLa cells in above noted conditions, normalized at 100% for the control wells. Data are the mean ± SD of at least three independent experiments.

The proliferation of HeLa cells reflects their ability to replicate themselves. It was evaluated at the 14th day after exposure to various concentrations of TDMQ20 (from 0 to 12 μM), by staining the adherent cells using the crystal violet dye which binds to proteins and DNA. Results are reported in Fig. 4. The number of HeLa cells in cultures treated with TDMQ20 drastically decreased with increasing drug concentration from 1.5 to 12 μM. At a concentration of TDMQ20 = 12 μM, HeLa cells were not detectable.

#### Anti-migration activity of TDMQ20 against HeLa cells. Wound healing assay (or “scratch assay”)

**Fig. 5:**
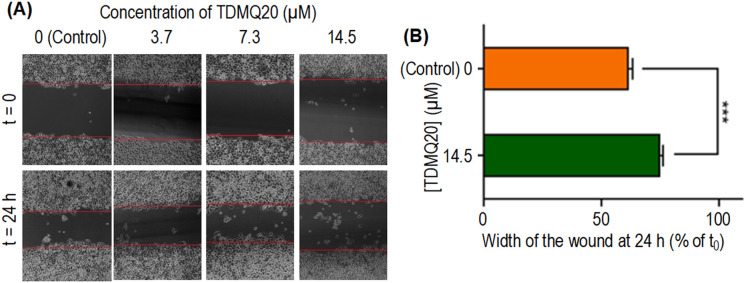
(A) Representative images of a wound healing assay of HeLa cell monolayers treated for 24 h with TDMQ20 at concentrations 0 (control), 3.7, 7.3 or 14.5 μM (B) histograms of the wound width of HeLa cells in above noted conditions, normalized at 100% for the control layer. Data are the mean ± SD of at least three independent experiments.

Cell migration is an essential process in all multicellular organisms and a hallmark of tissue development such wound repair. For cancer cells, cell migration is involved in several processes such as tumour invasion, neo-angiogenesis and metastasis.^25^ The wound healing (or scratch) assay is a method to measure two-dimensional cell migration. Then, migratory ability (motility) of HeLa cells in the presence of TDMQ20 (0–14.5 μM) was evaluated using the wound healing assay monitored by optical microscopy. Measurement after 24 h of the width of the “wound” created in a cell monolayer allows the quantification of the migration rate of the cells to close the wound. This method mimics cell migration during wound healing *in vivo*. Results are reported in Fig. 5.

The wound width in the absence of TDMQ20 was 61% at 24 h compared to its width at t_0_ (Fig. 5B, orange bar). After treatment by TDMQ20 14.5 μM, the wound width was 74% compared to t_0_ (Fig. 5B, green bar). So, *in vitro*, TDMQ20 at 14.5 μM inhibited by 33% the migration of HeLa cells to close the wound [(100 − 61) − (100 − 74)/(100 − 61) = 33%]. This result indicates that the drug exhibited a significant cell anti-migratory effect.

So, the antiproliferative activity of TDMQ20 in the colony-forming assay and its antimigratory activity in the “scratch assay” suggest that TDMQ20 should be able to inhibit tumour growth and metastasis.

### Mechanisms of activity of TDMQ20 on HeLa cancer cell lines

#### TDMQ20 promotes apoptosis

**Fig. 6:**
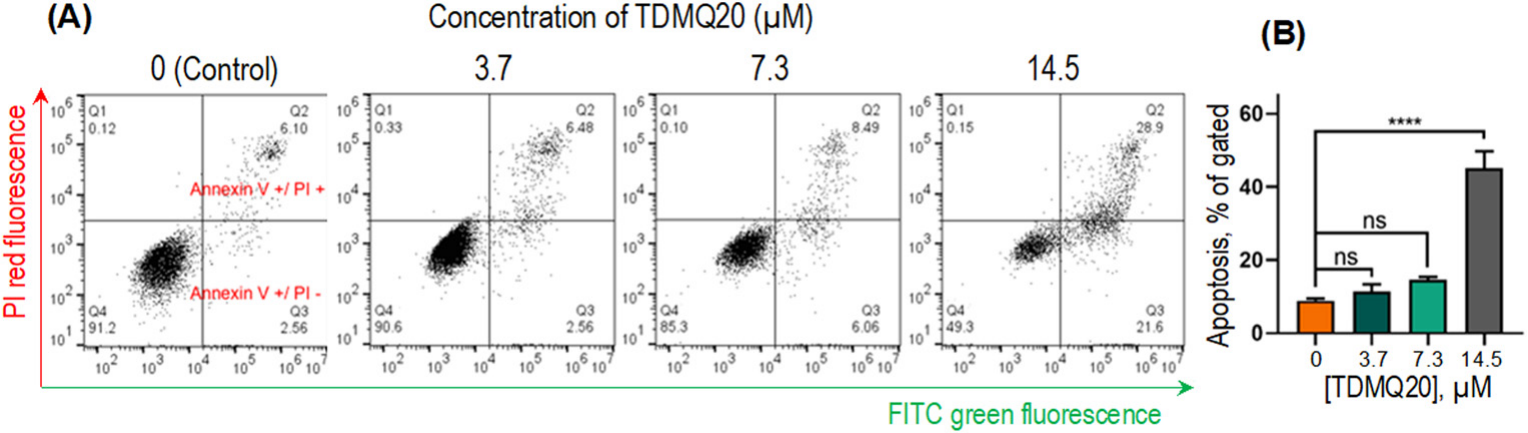
(A) Representative images of the pro-apoptotic activity of TDMQ20 on HeLa cells after 48 h of incubation, using annexin V-FITC/propidium iodide (PI) staining, and detection by flow cytometry. Necrotic cells (quadrant Q1), advanced apoptotic cells (Q2), early apoptotic cells (Q3) and non-apoptotic cells (Q4). (B) Histograms represent the proportion of apoptotic cells (Q2 + Q3); “ns” stands for *p* > 0.05, ****p < 0.0001 *vs.* untreated cells ([TDMQ20] = 0).

The pro-apoptotic activity of TDMQ20 in HeLa cells was evaluated after 48 h of incubation of HeLa cells with TDMQ20 at increasing concentrations (0–14.5 μM). Staining with annexin V-fluoresceine-5-isothiocyanate (FITC) was used for the detection of phosphatidylserine as a marker of early apoptosis (green fluorescence), and propidium iodide (PI) for detection of loss of nuclear membrane integrity as a marker of necrosis and late stages of apoptosis (red fluorescence). After staining, live cells show little or no fluorescence (annexin V−/PI−), early apoptosis cells show green fluorescence (annexin V+/PI−), late apoptosis cells and necrosis cells show red and green fluorescence (annexin V+/PI+). Cells were detected by flow cytometry. IDEAS software was used for acquisition of data and FlowJo v. X software was used for analysis of data. Results are reported in Fig. 6.

The total proportion of apoptotic cells, including early apoptosis (quadrants Q2) or late apoptosis (quadrants Q3) or necrosis (quadrants Q1), accounted for 51%, 15% and 9% when HeLa cells were treated with TDMQ20 at the concentration of 14.5 μM, 7.3 μM and 3.7 μM, respectively (Fig. 5B). So, TDMQ20 significantly induced the apoptosis of HeLa cells in a dose-dependent manner.

#### TDMQ20 induces overproduction of reactive oxygen species (ROS)

During aerobic metabolism, cells constantly generate reactive oxygen species (ROS) resulting from the reductive activation of dioxygen. The ROS generation plays an important protective and functional role in the immune system, and oxidative burst is responsible for anti-infectious activity of macrophages. However, when the generation of ROS overwhelms the cell natural defences, the production of the highly oxidant hydroxyl radical HO· induces random and potentially lethal cell injury. So, a balance must be struck between the relative abundance of ROS and cell antioxidants. Then, eukaryotic cells have developed complex systems to regulate the production and response to ROS, mainly based on superoxide dismutases (SODs), glutathione peroxidase, glutathione reductase, and catalase. Oxidative stress, defined as an excess of ROS production, has been linked to neurodegenerative diseases, cardiovascular diseases and many other pathologies, including cancer.^26–28^ At low to moderate levels, ROS act as signal transducers to activate cell proliferation, migration, invasion, and angiogenesis. In contrast, high levels of ROS are harmful to cancer cells and ultimately lead to cell death, and inhibit their metastatic spread (by inhibiting intravasation to enter the blood stream, that is necessary for tumour cells to generate metastases).^27^

**Fig. 7:**
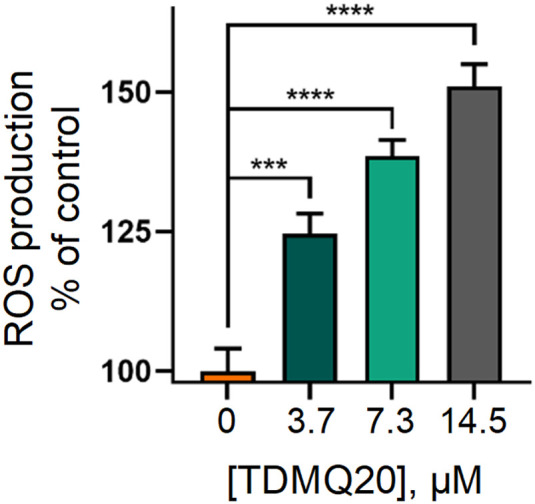
Fluorescence of DCF in HeLa cells incubated with TDMQ20 at 3.7 μM, 7.3 μM and 14.5 μM, as a measure of the percentage of ROS compared to untreated cells (control, [TDMQ20] = 0).

The most straightforward technique for measuring the production of reduced oxygen species in cells, is the use of the cell permeable fluorescent probe 2′,7′-dichlorodihydrofluorescein diacetate (H_2_DCF-DA). In the presence of ROS, predominantly H_2_O_2_, H_2_DCF-DA substrate is rapidly oxidized to 2′,7′-dichlorofluorescein (DCF) which is highly fluorescent, with excitation and emission wavelengths of 498 and 522 nm, respectively. The stained cells, whose fluorescence was an indirect measure of ROS level, were analysed by flow cytometry and analysed by GraphPad Prism 8 software.^29^ Results are reported in Fig. 7. The fluorescence of DCF in HeLa cells incubated with TDMQ20 at 3.7 μM, 7.3 μM and 14.5 μM, was 125%, 138% and 150% of the control cells, after 48 h of incubation with the drug. So, TDMQ20 induced a dose-dependent increase of ROS production in treated HeLa cells.

ROS are able to induce cell necrosis;^27^ they also stimulate events that lead to disrupt mitochondrial membrane potential, resulting in release of cytochrome c and activation of caspase-induced apoptosis.^26,28^

#### TDMQ20 induces collapse of the mitochondrial inner transmembrane potential (ΔΨm)

Since ROS level was increased in HeLa cells treated with TDMQ20, their potential damaging effect on the mitochondrial inner transmembrane potential ΔΨm was evaluated.

The direction of the mitochondrial membrane potential (with the interior of the organelle being negatively charged) allows to produce inward transport of cations and outward transport of anions, thus promoting accumulation of cations in the mitochondria and driving the synthesis of ATP. However, during apoptosis, the mitochondrial transmembrane potential (ΔΨm) decreases along with the opening of the mitochondrial permeability pores. Thus, ΔΨm is an essential parameter of the mitochondrial function that can be used as an indicator of cell health.^30^ The fluorescent membrane-permeant JC-1 dye exhibits potential-dependent accumulation in mitochondria, indicated by a green fluorescence (emission at 527–529 nm) for the monomeric form of the probe, which shifts to red (emission at 590 nm) with a concentration-dependent formation of red fluorescent J-aggregates in the cell. Therefore, the ratio of red/green fluorescence intensities is significant of mitochondrial integrity and function, and mitochondrial depolarization, signature of apoptotic cells, is indicated by a reduction in the red to green fluorescence intensity ratio.^31,32^ The higher is the ΔΨm, the more elevated is the red shift of the dye and, then, the red/green fluorescence intensity ratio.

**Fig. 8:**
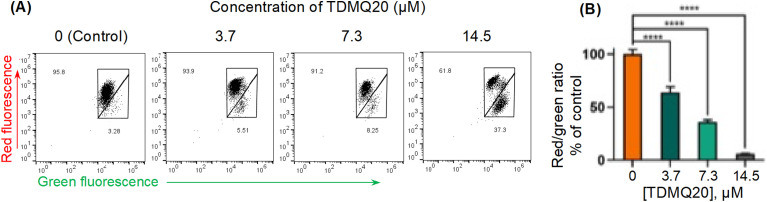
(A) Red and green fluorescence intensities of HeLa cells stained by JC-1, after 48 h of incubation with TDMQ20 at 0, 3.7 μM, 7.3 μM and 14.5 μM. Detection by flow cytometry. (B) Histograms represent the red/green fluorescence radio according to the applied dose of TDMQ20, as a percentage of the red/green ratio of untreated cells ([TDMQ20] = 0); ****p < 0.0001 *vs.* untreated cells.

This method was used to evaluate the mitochondrial membrane potential in HeLa cells incubated with various concentrations of TDMQ20 for 48 h, using staining with JC-1, and quantification by flow cytometry. Data analysis was carried out with the FlowJo v. X software. Results are reported in Fig. 8. The drastic decrease of the red/green fluorescence ratio while the concentration of TDMQ20 increased (Fig. 8B) indicates the collapse of the mitochondrial transmembrane potential, suggesting a drastic alteration of mitochondria significant of an apoptotic process.

## General discussion

TDMQ20 displayed various degrees of cytotoxic activity against the five tested cancer cell lines. This specific chelator of Cu(ii) was particularly efficient on the non-small cell lung carcinoma A549, the cervix cancer HeLa cells and the hepatocarcinoma HepG2, with IC_50_ values in the range 14–16 μM, significantly lower than that of the reference drug 5-FU. This effect is dose-dependent. In addition, its selectivity for cancer cells compared to non-cancer HaCaT cells is significantly higher than that of 5-FU. Moreover, TDMQ20 at 3–15 μM inhibits in a dose-dependent manner, migration of HeLa cells *in vitro*, in the colony-forming assay and in the wound-healing assay, suggesting that this drug could prevent cancer cells proliferation and metastasis *in vivo*.

Preliminary investigations on the mechanism of action of TDMQ20 on HeLa cells *in vitro* indicate that TDMQ20 (i) enhances ROS production, (ii) drastically decreases the mitochondrial transmembrane potential ΔΨm, and (iii) induces apoptosis. In fact, the concomitance of these three phenomena is expected, because they are linked together. Mitochondrial outer membrane permeabilization and cytochrome c release promote caspase activation and execution of cell apoptosis. Among the first targets of activated caspases are the permeabilized mitochondria themselves, leading to the disruption of electron transport, the loss of ΔΨm, the decline in ATP levels, the production of ROS (due to electrons in the respiratory chain losing their way), and the loss of mitochondrial structural integrity. So, reduction of ΔΨm appears to be a key event and marker during apoptosis.^33,34^

In human body, whatever the oxidation state, total iron is 40 times more abundant than copper (0.006% and 0.0002% of body weight, respectively). Iron is roughly 500 times more abundant than copper in human whole blood, a fluid particularly rich in iron, the concentrations being close to 8 mM and 15 μM, respectively. So, the highest difference in Fe/Cu concentrations is 3 orders of magnitude. However, as emphasized above, the affinity of TDMQ20 for Cu(ii) is higher than that of Fe or other common biological metal ions,^21,22^ by (at least) 13 orders of magnitude {log *K*_app_ [Cu(ii)-TDMQ20] = 16.5 and log *K*_app_ [Fe(ii)-TDMQ20] < 3}. Then, complexation of iron is expected to be negligible compared with that of copper. Because of this, one can consider that the involvement of copper coordination in the anti-cancer and pro-apoptotic mechanism of action of TDMQ20 is a reasonable proposal. Note that many metabolic pathways are interdependent, and this applies in particular to iron and copper. For example, the major Cu transporter ceruloplasmin exhibits a ferroxidase activity that promotes Fe^2+^ oxidation during iron loading into ferritin. So, an indirect role of TDMQ20 on iron metabolism cannot be ruled out.

The fact that TDMQ20 is active in three different pathologies as different in appearance as Alzheimer's disease (AD),^21,23^ Wilson's disease (WD)^35^ or particular cancers can be questioned. In fact, all these three pathologies have in common the fact the regulation of copper is perturbated. In AD, copper is accumulated in amyloid plaques, in WD copper excess is not excreted due to mutations on a copper-carrier and in cancer copper is playing a key role in metastasis generation. In all three cases, the activity of TDMQ20 should be related to its capacity to selectively chelate copper ions with its suitable tetradentate structure leading to a stable copper(ii) complex. Consequently, there are factual reasons to develop such specific copper ligand on three different diseases. Future clinical developments will be necessary to find which therapeutic domain will be best tackled by TDMQ20.

## Experimental

### Materials and methods

TDMQ20 was synthesized according to the published procedure.^21^ Dulbecco's modified eagle medium (DMEM), fetal bovine serum (FBS), phosphate buffer saline (PBS, 1X), trypsin-EDTA (0.25%) and penicillin–streptomycin–amphotericin B solutions were purchased from GIBCO (Grand Island, NY, USA). 3-(4,5-Dimethylthiazol-2-yl)-2,5-diphenyltetrazolium bromide (MTT) was purchased from Innochem (Beijing, China), 5-fluorouracil (5-FU) was purchased from Aladdin (Shanghai, China), dimethyl sulfoxide (DMSO) was purchased from Sigma-Aldrich (Shanghai, China). Commercially available chemicals were used as purchased, without purification. All experiments were carried out at least in triplicate and standard deviations were calculated by GraphPad Prism 8. The bar charts represent mean values ± SD, and images are representative of one experiment. Statistical analyses were performed using one-way ANOVA. Differences with *p* > 0.05 were considered not significant (ns), *p < 0.05, **p < 0.01, ***p < 0.001 and ****p ≤ 0.0001.

### Titration of TDMQ20 by Fe^II^(SO_4_)_2_(NH_4_)_2_·6H_2_O

The titration of TDMQ20 was achieved in Hepes buffer (50 mM, pH 7.4, treated with Chelex® resin) and monitored by UV-vis. spectrometry, as reported in ref. 21, except that TDMQ20 and Fe(ii) salt solutions were carefully degassed and kept under argon. The mixture TDMQ20 + Fe^II^ salt was carried out in a UV-vis. Quartz cuvette maintained under an argon atmosphere. The concentration of TDMQ20 in the cuvette was 20 μM (final volume: 1.5 mL). Aliquots of a solution of Fe(SO_4_)_2_(NH_4_)_2_·6H_2_O were added, corresponding to 5, 10, 20, 30 molar equivalents with respect to TDMQ20, under gentle magnetic stirring (final iron concentration = 600 μM). Total volume of Fe(SO_4_)_2_(NH_4_)_2_·6H_2_O solution added was 30 μL (Δ*v* in the cuvette = 2%). The UV-visible spectrum of the mixture was recorded 2 min after addition of each iron aliquot, using a Cary 3500 spectrophotometer from Agilent (France). The experiment temperature was 24 °C.

### Cell lines

Cancer cell lines, A375 (melanoma), A549 (lung), HeLa (cervical), HepG2 (liver), and HaCaT (human immortalized keratinocytes) were obtained from the Cell Bank of the Chinese Academy of Science (Wuhan, China). COLO829 (melanoma) cells were obtained from American Type Culture Collection (ATCC).

### Drug solutions

Mother solutions of TDMQ20 and 5-FU were prepared extemporaneously at 100 μM (which is the maximum concentration used in the experiments) by dissolving a weighted amount of each drug in DMEM. The used molecular weights were 449.93 g mol^−1^ or 130.08 g mol^−1^ for TDMQ20 (prepared according to ref. 21) and 5-FU (purchased from Aladdin, China), respectively.

### Cytotoxicity evaluation

For evaluation of the cytotoxicity, 5000 cells were seeded in each well of the 96-well plates, incubated at 37 °C, 5% CO_2_ humidified environment for 24 hours, and various concentrations of drugs were then added. The MTT assay was carried out after 48 h of incubation of different concentrations (100, 50, 25, 12.5, 6.3, or 3.1 μM) of TDMQ20 by adding 100 μL of a 0.5 mg mL^−1^ of MTT dissolved in DMEM FBS-free. Absence of TDMQ20 was the control. The plates containing MTT were incubated at 37 °C, in a 5% CO_2_ humidified atmosphere for 4 h. After this period, the MTT solution was removed and 150 μL of DMSO was added to solubilize the purple formazan crystals formed by metabolically active cells. Survival was analysed spectrophotometrically at 570 nm, using a Multi-Mode Detection Platform (SpectraMax@ Paradigm@). IC_50_ represents half value of maximal inhibitory concentration. 5-FU was used as a positive control.

### Colony formation assay

A total of 1000 Hela cells per dish was seeded into 3.5 cm dishes. After attachment, cells were pretreated with various concentrations (12, 6, 3, 1.5, 0 (control) μmol L^−1^) of TDMQ20. During the 14 days of the experiment, the medium was refreshed every 2 days. After 14 days culturing, cells were washed with PBS, fixed with 100% methanol for 30 min, and then stained with 0.1% crystal violet for 30 min. The plates were air-dried, and pictures of the visible colonies were taken under an optical microscope, Olympus. The Image-Pro Plus 6.0 software was used for picture analysis.

### Evaluation of TDMQ20 in the wound healing assay

HeLa cells were seeded and allowed to attach to the 6-well plate for live-cell imaging overnight. Then, cells were washed with a fresh DMEM medium and incubated with TDMQ20 at various concentrations (14.5, 7.3, 3.7, 0 μM) for 24 h. Images were captured under 100× magnification using a fluorescence microscope (Olympus, Japan).

### Pro-apoptotic activity of TDMQ20

HeLa cells were seeded into 10 cm culture dishes. After overnight attachment, cells were treated with TDMQ20 at various concentrations (14.5, 7.3, 3.7, 0 μM) for 48 h. Cells were harvested and stained with FITC-annexin V (Beyotime) according to the manufacturer's instructions. Finally, cells were stained with propidium iodide (Beyotime) and assessed using Flowsight flow cytometer (Merck, U.S.A.), IDEAS 6.2 software, GraphPad Prism 8 and FlowJo v.X softwares.

### ROS production in HeLa cells, induced by TDMQ20

The extracellular ROS levels were determined using 2′,7′-dichlorodihydrofluorescein diacetate probe (H_2_DCFDA, Beyotime), following the manufacturer's protocol. Briefly, Hela cells were treated with TDMQ20 at 14.5, 7.3, 3.7, or 0 μM for 48 h. Then the cells were enzymatically detached using trypsin-EDTA, washed in PBS, resuspended in the staining solution prepared according to manufacturer's instructions (1 mL of H_2_DCFDA per well, final concentration of H_2_DCFDA = 10 μM), and incubated at 37 °C for 30 min. During the experiment, samples were kept in the dark, to avoid light-induced ROS production. Finally, the stained cells were analysed by flow cytometry using Flowsight flow cytometer (Merck, U.S.A.), IDEAS 6.2 and GraphPad Prism 8 softwares.

### Measure of the mitochondrial potential membrane (ΔΨm) in the presence of TDMQ20

HeLa cells were seeded into 10 cm culture dishes and allowed to attach overnight. Then, the cell cultures were treated with TDMQ20 at 14.5, 7.3, 3.7 or 0 μM and incubated for a further 48 h. The ΔΨm was assessed using JC-1 according to manufacturer's instructions. Finally, the stained cells were analysed by flow cytometry using Flowsight flow cytometer (Merck, U.S.A.), IDEAS 6.2 software, GraphPad Prism 8 and FlowJo v.X software.

## Conclusions

The reported results indicate that TDMQ20 is more active than 5-fluorouracil (5-FU) on non-small cell lung carcinoma (A549), cervix cancer HeLa and hepatocarcinoma HepG2 cell. In addition, the safety window of TDMQ20 is higher than that of 5-FU. The mechanism of cytotoxicity and antiproliferative activity of TDMQ20 involves the intracellular production of reactive oxygen species generating drastic mitochondrial damages and the induction of tumor cell apoptosis.

## Author contributions

Conceptualisation, methodology, supervision and funding acquisition: YL and BM, investigation: YG and MN, project administration: YL, validation: YL, AR and BM, writing original draft: AR. All authors have read and agreed to the submitted version of the manuscript.

## Conflicts of interest

There are no conflicts to declare.
